# Challenges and Approaches in Microbiome Research: From Fundamental to Applied

**DOI:** 10.3389/fpls.2018.01205

**Published:** 2018-08-17

**Authors:** Chrysi Sergaki, Beatriz Lagunas, Ian Lidbury, Miriam L. Gifford, Patrick Schäfer

**Affiliations:** ^1^School of Life Sciences, University of Warwick, Coventry, United Kingdom; ^2^Warwick Integrative Synthetic Biology Centre, University of Warwick, Coventry, United Kingdom

**Keywords:** microbial community, root interactions, cropping systems, gnotobiotic, omics, microbial function

## Abstract

We face major agricultural challenges that remain a threat for global food security. Soil microbes harbor enormous potentials to provide sustainable and economically favorable solutions that could introduce novel approaches to improve agricultural practices and, hence, crop productivity. In this review we give an overview regarding the current state-of-the-art of microbiome research by discussing new technologies and approaches. We also provide insights into fundamental microbiome research that aim to provide a deeper understanding of the dynamics within microbial communities, as well as their interactions with different plant hosts and the environment. We aim to connect all these approaches with potential applications and reflect how we can use microbial communities in modern agricultural systems to realize a more customized and sustainable use of valuable resources (e.g., soil).

## Introduction

Soil is considered as one of the most diverse habitats on Earth containing billions of bacteria and millions of fungi (comprising thousands of taxa), as well as larger organisms such as nematodes, ants, or moles (Bardgett and van der Putten, 2014). Recent advances in high throughput sequencing techniques and the increasing number of microbial culture libraries are contributing to show an expanded version of the tree of life dominated by bacterial diversification (Hug et al., 2016). This enormous diversity is driven by the ability of microbes to perform lateral gene transfer across disparate phylogenetic groups (McDonald and Currie, 2017). Moreover, microbial communities are built on high numbers of individuals for each species (Robbins et al., 2016), that can quickly proliferate and have high mutation rates (in the range of 10^−4^ in *E. coli*) (Kibota and Lynch, 1996; Boe et al., 2000; Denamur and Matic, 2006) as compared to higher organisms like humans [10^−8^] (Kuroki et al., 2006; Xue et al., 2015). These characteristics increase the diversification of microbes and microbial communities, where individual microbes of the same species could potentially bear different genetic endowments and thus functional characteristics.

Soil microbes play key roles in the cycling of nutrients such as nitrogen or phosphorus as well as providing plant protection against biotic and abiotic stress (Bender et al., 2016; Lladó et al., 2017). Intensive agriculture has contributed to increases in crop yields but at the same time it has had detrimental effects on the physical and biological properties of soils (Pimentel et al., 1995; Bouwman et al., 2009). In intensively managed agricultural systems, the application of fertilizers can compensate for a loss of soil fertility, while tillage disrupts microbial communities (Johnson et al., 1997). This is particularly relevant in the light of current crop production systems with the degradation of more than one half of the global agricultural land while we face massive challenges associated with the disturbance of nitrogen and phosphorous cycles. This situation is very likely to worsen under the prospect of the climate change (Rockström et al., 2009; Yuan et al., 2018). As a consequence the United Nations has suggested the re-introduction of sustainable land management practices to minimize land degradation (Sanz, 2017). These practices include crop diversification, use of local adapted species or intercropping in order to maintain soil fertility, carbon sequestration, and nutrient cycling as well as to control soil erosion (Sanz, 2017). Interestingly, these procedures also enhance general soil disease suppression (Weller et al., 2002; Bonilla et al., 2015). In addition, sustaining microbial community diversity, structure and composition can help to support ecosystem functions, e.g., by regulating nutrient cycles.

During the last decade, microbiome research has modified our perception on the complexity and structure of microbial communities. However, we are only just starting to understand the organization of such complex communities, the interdependencies among themselves and with the biotic (e.g., plant) and abiotic (e.g., edaphic) environment. The increasing need for alternative experimental approaches, as well as the development of new tools has provided new insights into our understanding of the dynamics that occur within the microbiomes and their interaction with host organisms (Goodrich et al., 2017). In studying the human microbiome, the complexity of microbial interactions and the importance of analyzing them separately for each individual has already resulted in novel therapies. Considering the unique microbiome signature of each host, we could move toward a personalized application of microbiome, where we would be able to handle each case independently and better tailor the microbiome to the host’s needs, thus increasing the efficiency of the treatment and the potential of the host (Human Microbiome Project Consortium, 2012). Such “personalized” microbiome approaches would be particularly facilitated by the genetic uniformity of host genotypes of a given crop plant species in the field. Similar to human microbiome studies, there have been efforts to understand the complexity of soil and plant microbiomes (Bulgarelli et al., 2012; Lundberg et al., 2012) and to fuel new innovations in sustainable crop production as part of the next green revolution (Jez et al., 2016). However, to exploit the full potential of microbiomes, we require the development of new analytical strategies to comprehend the array of functional capabilities of microbial communities (Bashiardes et al., 2018). The importance of maintaining a diverse and well-balanced microbiome at the plant–soil interface is vital in crop production. Any microbiome applications, however, have to focus on improving key determinants of crop production such as nutrient availability, soil fertility and soil health (Syed Ab Rahman et al., 2018). In this respect, the key challenge is to transfer lab-generated knowledge to the field. In addition to unraveling the structure of the plant/soil microbiome (Schlaeppi and Bulgarelli, 2015), it especially requires us to connect microbial community dynamics with microbiome functioning (Sánchez-Cañizares et al., 2017). In this review we present the challenges and latest efforts that have been made in order to advance our understanding of the different dimensions of microbiomes (e.g., structure, dynamics) and how it affects plants. We further introduce future approaches to access the full potential of the soil microbiome, including beneficial microbes, in improving crop production.

## The Expansion of Microbiome Research in the “Omics” Era

The reduction in sequencing costs in addition to advances in sequencing technologies and increased computational power has facilitated an overwhelming number of soil and rhizosphere-related microbiome studies (Prosser, 2015; Fierer, 2017). Researchers commonly employ three main types of sequencing: (1) metataxonomic primer-based amplicon sequencing, which focuses on the amplification of specific regions of ubiquitous genetic markers, usually 16S rRNA (Bacteria and Archaea) or the intergenic spacer (ITS) region (Eukaryotes), (2) shotgun sequencing of the entire genomic or transcriptomic information within a given sample (metagenomics and metatranscriptomics) (3) detection of separated and fragmented proteins (metaproteomics), usually by combining liquid chromatography mass spectrometry (LC MS-MS), and (4) detection of metabolites, normally through MS or nuclear magnetic resonance (NMR) (metabolomics). The technical limitations of such metaomics and amplicon sequencing, such as sampling errors, primer or processing biases, computational power and adequate analytical algorithms have been extensively discussed in several comprehensive reviews and will not be discussed here (Hirsch et al., 2010; Carvalhais et al., 2012; Pinto and Raskin, 2012; Scholz et al., 2012; Temperton and Giovannoni, 2012; Tkacz and Poole, 2015). Encouragingly, major progress has been made in alleviating these limitations, but even with “perfect” metaomics techniques, many conceptual limitations, such as extrapolating accurate information from metaomics datasets to draw meaningful conclusions, still exist and require careful experimental design (Prosser, 2015). For example, mis-annotation of genes in datasets is a major limitation on extrapolating data from omics datasets (Lidbury et al., 2014; Fierer, 2017). In addition, particularly with regards to metagenomics, we tend to focus on genes with known functions and ignore a whole suite of genetic information that harbors the potential to perform novel functions, particularly with regards to genes that encode ecologically important extracellular proteins (Christie-Oleza et al., 2015).

### New Efforts Toward the Characterization of Complex Microbial Communities

One clear benefit of amplicon sequencing is that multiple samples can be processed simultaneously in one sequencing experiment allowing for increased spatiotemporal resolution and the ability to test multivariate factors. To this end, amplicon sequencing has been invaluable in determining general patterns of microbial diversity within the plant microbiome (Bulgarelli et al., 2012; Lundberg et al., 2012; Peiffer et al., 2013; Tkacz et al., 2015). Amplicon sequencing is now frequently employed with more sophisticated techniques, for example exometabolic profiling of plant exudates (Badri et al., 2013; Zhalnina et al., 2018), multi-generation plant trait selection experiments (Haney et al., 2015; Panke-Buisse et al., 2015), crop mutant line experiments (Senga et al., 2017), and microscopy (Rybakova et al., 2017) to help answer specific questions about plant-mediated bacterial recruitment and functioning in the rhizosphere. Indeed, using 16S rRNA gene data has also revealed that members of the “rare” biosphere are actively recruited in the rhizosphere suggesting that they may play an important role despite their low abundance (Dawson et al., 2017). Furthermore, Bulgarelli et al. (2015) suggested the need to combine alternative markers to 16S rRNA, such as 18S rRNA or internal transcribed spacers (ITSs), in order to access and characterize a broader range of microbes and to get a more representative picture of the microbial diversity and structure. Indeed, metagenomics or metatranscriptomics can partially alleviate this problem by sequencing all the genomic content in a sample simultaneously (Chaparro et al., 2014; Bulgarelli et al., 2015).

### Challenges and New Efforts in Assigning Function to Microbes

Beyond determining microbiome structures under different environments, it is particularly challenging to assign a function to individual microbiome members or groups and we tend to consider each microbial group as a functional group. However, even species within a particular genus can have completely different lifestyles - from pathogen to mutualist depending on the environment (Hacquard et al., 2016; Hiruma et al., 2016), or due to horizontal transfer of specific functional genes (Qiu et al., 2009). This variability can lead to dramatic changes in microbial phenotypes of desired traits, such as phosphorus mobilization (Lidbury et al., 2016). More sensitive methods to characterize the microbiome beyond the genus level are needed, along with a better functional characterization of each species, which would require large scale/high throughput techniques (Schlaeppi and Bulgarelli, 2015). With the rate at which technology advances, combining, e.g., computational/modeling methods are very promising. For instance, there is a transition from metagenomics to metaphenomics, which combine the product of the metagenome (or “expressed functions encoded in microbial genomes”) with the environment, taking all the parameters into account that may influence the dynamics of the interactions within the community and the environment (Jansson and Hofmockel, 2018). In this respect, metagenomics can be potentially powerful and provide information about the broad functional capabilities (e.g., secondary metabolite production or carbohydrate utilization) (Bulgarelli et al., 2015) or about specific gene sets (e.g., metabolic pathways) of the microbiome (Wang et al., 2015; Ofaim et al., 2017). With new analytical methods, we can also gain deeper insights into the specific taxa responsible for harboring key functional traits (Prosser, 2015). With the development of easy-to-use commercial kits, extracting DNA from a given sample is straightforward. However, DNA only gives the functional “potential” of a microbial community. Furthermore, particularly in soils, the vast majority (>90%) of microbial biomass (or genetic information) is inactive or dormant (for a comprehensive review, see Fierer, 2017). Nevertheless, this number is likely to drop significantly in the rhizosphere and root, due to the step-wise selection of microbes by plant-mediated factors meaning more microbes are metabolically active in these niches (Bulgarelli et al., 2013). Efforts have been made to extract RNA from rhizosphere samples to look at those microbes that are metabolically active and reveal which genetic pathways they are inducing in response to plant and microbial stimuli (Turner et al., 2013b; Chaparro et al., 2014; Yergeau et al., 2014). Therefore, many studies combine the enrichment of 13C-labeled CO_2_ with metatranscriptomics to identify microbes responding to plant exudates to improve our understanding on the interactions between microbiome and plant host (Haichar et al., 2016; Lueders et al., 2016).

Unlike, the shorter turnover times of RNA, which reduces the simplicity and robustness of sampling efficiency (Prosser, 2015), proteins, especially exoproteins, are more stable in the environment (Armengaud et al., 2012). Thus, sampling is methodologically easier prone to sampling errors/artifacts. Metaproteomics also provides an exciting opportunity in omics research as it gives a profile of expressed proteins, and hence, (metabolic) activities in a given sample (Heyer et al., 2015). In turn, exoproteomics or exometaproteomics, enriches for the more ecologically important proteins that are involved in nutrient acquisition and microbial–microbial and microbial–host interactions (e.g., extracellular hydrolytic enzymes and transporter systems) (Armengaud et al., 2012; Lidbury et al., 2016). However, there are several drawbacks to metaproteomics, particularly a requirement for sufficient starting material (sometimes up to 100 g of soil is needed) (Johnson-Rollings et al., 2014), as well as the accurately assignment of peptides detected to the correct proteins, which relies on a comprehensive databases (metagenome) and sufficient computational power (Muth et al., 2013, 2016; Timmins-Schiffman et al., 2017). Perhaps this is why meta(exo)proteomics has not been extensively utilized in rhizosphere research, in comparison to studies of other less complex microbial niches, e.g., seawater and anaerobic digesters (Sowell et al., 2009; Williams et al., 2012; Heyer et al., 2015), and enriched chitin-degrading sandy soil samples (Johnson-Rollings et al., 2014). Similarly, this approach can be very powerful for identifying the major extracellular enzymes involved in phosphate mobilization within the rhizosphere (Lidbury et al., 2016; Lidbury et al., unpublished results).

### Efforts to Isolate, Characterize, and Use Microbial Strains in Synthetic Communities

As the major rhizobacterial phyla (Actinobacteria, Proteobacteria, Bacteroidetes, and Firmicutes) are amenable to cultivation, a number of new studies have reverted to extensive isolation efforts followed by genome sequencing and phenotypic characterisation (Bai et al., 2015; Mauchline et al., 2015; Levy et al., 2018). These are often combined with the reconstruction of synthetic communities to determine keystone species and patterns of recruitment in the rhizosphere (Bai et al., 2015; Niu et al., 2017). Reconstruction of microbial communities can help to identify microbe–microbe interactions that have an effect on plant growth (Hartman et al., 2017). The advantage of the isolation approach is that sequencing and assembling of individual genomes is much simpler and usually provides a higher resolution of data than assembling metagenomes collected *in situ*. More, the isolation approach is a sophisticated tool to functionally validate isolates within a community and/or their interaction with host plants (Levy et al., 2018). Any isolates exhibiting either novel or improved functionality can easily be deployed for further investigation to identify the precise molecular mechanisms and associated rate kinetics of key enzymes. For example, studying the transcriptomic or proteomics response of individual bacterial or fungal isolates to the plant microbiome or associated nutrient stresses has provided useful information on the genes involved in potentially important plant growth promotion (PGP) processes and recruitment of beneficial microbes (Mauchline et al., 2006; Fernández et al., 2013; Lidbury et al., 2016; Martino et al., 2018). This approach can also be particularly useful for the discovery of novel traits associated with PGP activities mediated by microbes (Bruto et al., 2014). Since the *in vitro* screening methods for PGP traits do not necessarily reveal phenotypes associated with plants, the use of genomic screening tools could provide a fast, large scale screening while encouraging the discovery of novel PGP traits/genes (Finkel et al., 2017). Combining these methods with complementary molecular approaches, such as mutagenic and bio-reporter expression systems (Wetmore et al., 2015; Cheng et al., 2016; Pini et al., 2017) will uncover the role of these PGP traits/genes and improve our predictions about the mechanisms driving interactions within plant microbiomes.

## Experimental Set Ups to Study Microbiomes

### The Need to Understand the Interactions

Plants can affect the structure of their root microbiome in favor of beneficial microbes and against pathogens or other deleterious microbes (Berendsen et al., 2012; Bulgarelli et al., 2015). In turn, microbes can also manipulate the host for their own benefit, e.g., altering host metabolism (Jones and Dangl, 2006; Engelstädter and Hurst, 2009; Hacquard et al., 2015; Levy et al., 2018). In addition, the abiotic environment (e.g., edaphic factors) influences both the plant and microbial communities, further enhancing the complexity of ecosystems (Bakker et al., 2014). Network analyses have shown the importance of “microbial hubs,” which are strongly interconnected microbial taxa that severely influence communities and that are thought to be a key to understand microbiome dynamics, and the effect of single microbes on the structure of microbial communities (Agler et al., 2016). For instance, Niu et al. (2017) created synthetic communities using seven representative bacterial strains from the three most abundant phyla obtained from maize roots. Employing this simplified community, they aimed to uncover mechanisms that determine the dynamics of this system. Interestingly, the removal of one strain led to the complete collapse of the community, highlighting the importance of individual members of the microbiome (Niu et al., 2017). It further suggests that small or even subtle changes can lead to significant effects on microbiome structures. Therefore, deciphering underlying inter-microbial dynamics driving community structures can be key in validating stable synthetic communities. There are new efforts to analyse those complex interactions and establish reliable systems that can (i) overcome the soil ecosystem complexity and (ii) build our fundamental knowledge of microbial and plant–microbe interactions.

### Advances to Overcome Soil Ecosystem Complexity

Since the revelation of the *Arabidopsis thaliana* core root microbiome, which gave more detailed insights into plant microbiome structures (Bulgarelli et al., 2012), numerous studies have collectively highlighted the importance of microbiomes in ecosystem functioning (Agler et al., 2016). Furthermore, the isolation and characterisation of microbial species together with the development of defined gnotobiotic systems (Lebeis et al., 2015; Finkel et al., 2017) allows the targeted functional characterisation of individual members of plant microbiomes (Bai et al., 2015). Gnotobiotic systems, in particular, have been recognized as being essential for microbiome research as they allow to distinguish between the effect of microbes or microbial combinations and the environment (by applying defined conditions) on plant phenotypes. By revealing individual processes in multicomponent plant–microbe–environment interactions, it gives the possibility to associate genotypes with phenotypes. However, the reduced complexity of such systems as well as the “artificial” or *de novo* assembly of microbiomes can prevent the recapitulation and, hence, full functionality of natural systems (Vorholt et al., 2017).

Hartman et al. (2017) presented a different way of microbe application as an effort to understand complex interactions within microbiomes. They isolated microbes from the roots of *Trifolium pratense* and chose one representative strain from each of the four most abundant microbial groups (OTUs) to inoculate sterile microcosms. They reported a negative effect of *Flavobacterium* on the growth of *Trifolium*, which was alleviated in the presence of either of the three other bacterial representatives from *Pseudomonas*, *Janthinobacterium*, and *Microbacterium*. Interestingly, none of the three bacteria affected the abundance of the *Flavobacterium* in the synthetic community. Therefore, the negative activity of *Flavobacterium* was somehow “buffered” by *Pseudomonas*, *Janthinobacterium*, and *Microbacterium* (Hartman et al., 2017). Such a reductionist approach can reveal new “keystone” players in regulating microbiome function and its interaction with the plant.

All these analytical approaches have greatly benefitted from the advancements in computational analyses and machine learning and their significance for the development of microbiome research (Knight et al., 2018). COREMIC, for example, is a bioinformatics tool that allows the generation and confirmation of hypothetical models, by associating microbes with certain plants or habitants using existing databases (Rodrigues et al., 2018). As for other omics-based analyses, the need for reproducibility as well as the development of “golden standards” to improve consistency and comparability of experiments have been particularly highlighted (Knight et al., 2018). Furthermore, network analyses have equipped microbiome research with sophisticated tools that can analyse and explain the complexity of microbial communities (Adair and Douglas, 2017; Wang H. et al., 2017). While network analyses often build the basis in revealing the function of microbial taxa and the nature of microbial interactions (Poudel et al., 2016), it has certain limitations in identifying synergistic, additive and antagonistic effects. As a result, key functions of certain low abundant microbes might be underestimated or not even recognized (Shade et al., 2014; Shi et al., 2016). The ultimate aim of these efforts, the identification of interactions within microbial communities on plants as a result of inter–microbial communication, therefore requires sensitive tools uncovering correlative interactions that can be verified in biological assays.

### Advances in Fundamental Research on Microbe–Microbe and Plant–Microbe Interactions

Besides comprehending the complexity of microbial communities and interactions, there is the need to uncover basic regulatory (communicative) principles of interactions that can inform experimental design. Recently, cytology-based systems have been developed to study microbial interactions. Hennessy et al. (2017) established a microplate reader-based system, to quantify the activities and interactions between living microbes. This method represents a potential high-throughput screen by using live imaging of fluorescing metabolites and microbial growth to identify and trace the expression of certain genes in defined microbial communities. In their study, they measured the rate of the production of fluorescent metabolites of *Pseudomonas fluorescens* in response to the presence of *Fusarium graminearum*, as an indicator for their interaction (Hennessy et al., 2017). Alternatively, Massalha et al. (2017) published recently a microfluidics-based system for *in vivo* imaging of plant root–microbe interactions. Using a transparent chamber, they could record root zone preferably colonized by a fluorophore-tagged microbe. By adding a second microbe to the system they were able to study microbe–microbe interactions in real time. Despite their minimalistic set-up, such studies reveal fundamental insights into basic principles that shape microbe–microbe and root–microbe interactions. In this respect, the application of transparent soil represents an innovative approach to study and live image microbes on plant roots in an environment which mimics different soil textures (Downie et al., 2012). It allows to detect processes driving the distribution of microbes in bulk substrate along the root (Downie et al., 2014) and study the effects of major root pests such as nematodes on microbe/community behavior (O’Callaghan et al., 2018).

In addition to bacterial and fungal microbiomes, soil and plant processes are directly influenced by other organisms including viruses, archaea, nematodes, and insects. Viruses play a very important role in soil biochemical processes and act as gene reservoirs for horizontal gene transfer, although their function is not completely understood (Pratama and van Elsas, 2018). Similarly, Archaea and nematodes significantly contribute to microbiome diversity and in interaction with other microbes to soil-plant processes and ecosystem functioning (Adam et al., 2017; Castillo et al., 2017; Elhady et al., 2017). In this respect, Benítez et al. (2017) has given a very interesting insight into plant–microbe–insect interactions. They reported that soil microorganisms can affect aboveground interactions between plants and insects, by modulating the release of plant volatiles (Pineda et al., 2015; Beck and Vannette, 2017; Benítez et al., 2017). These studies indicate that we need more comprehensive, holistic studies on multitrophic interactions in order to understand which edaphic and biotic factors determine the structure and, hence, function of soil and plant microbiomes structures.

Exploiting the full potential of microbes and microbial communities will depend on expertise from different fields. In addition to improving our understanding of complex plant–microbe and multitrophic interactions using plant biology and microbiology-based approaches, we need to develop new ecological systems with growing complexity. Most critically, in order for this knowledge to be successfully transferred to agriculture it is essential to understand the impact of various farming practices on the microbiome and how this is translated to plant health and, thus, crop productivity. In addition, it is necessary to test microbial community function in a highly complex and diverse system (e.g., field), bridging the gap between the lab and the farm.

## Bridging the Lab-Field Gap

### Limitations on the Experiments Performed in Controlled Conditions (The Lack of Context)

Increasing evidence is showing that plant–microbe interactions can be beneficial or detrimental for either the host or microbial symbiont depending on the balance of associated biotic and abiotic factors. Whilst, experiments involving pairwise interactions under controlled conditions have increased our knowledge about gene and metabolite expression profiles involved in plant–microbe interactions, these experiments give us little information about microbial function in a natural ecosystem (de Boer, 2017). Although this was stated by de Boer (2017) for fungal–bacterial interactions it is applicable to many other interactions (even to those such as plant–rhizobia or plant–mycorrhizal interactions). For example, even some species of plant-growth promoting arbuscular mycorrhizae fungi (AMF) have been shown to inhibit plant growth under certain conditions, e.g., low light, low temperature or phosphorous (P) availability (Smith and Smith, 1996; Johnson et al., 1997). In addition, AMF activity can also be suppressed by the soil microbiota (Svenningsen et al., 2018) highlighting the practical need for field experiments to fully understand microbe behavior.

The main reason for the existence of this lab-farm gap is that lab studies generally do not capture the complexity of microbe–microbe interactions that occur in a natural setting. However, it is widely known now that microbial communities and plant–microbe interactions are highly dependent on the entire ecosystem (Bulgarelli et al., 2013; de Boer, 2017; Lewis et al., 2018). For instance, host genotypes have been shown to shape plant microbial communities (Bulgarelli et al., 2013; Horton et al., 2014) and a genome wide association study (GWAS) analysis revealed that both bacterial and fungal communities are structured by the same host biological processes (such as defense response or signal transduction). However, different genes seem to be involved in the interaction (Horton et al., 2014) and microbial communities are further fine-tuned during plant development according to host requirements (Chaparro et al., 2014). Host-dependent control of the microbial community is likely controlled by the flow of organic compounds from the root to the rhizosphere (rhizodeposition) (Chaparro et al., 2014; Baran et al., 2015) which has been shown to attract beneficial microbes and refrain pathogen attack. The legume-rhizobia symbiosis is an elegant example of rhizodeposits selecting for beneficial microbes. In response to low nitrogen, the host releases flavonoid compounds that initiate the molecular dialog with nitrogen-fixing rhizobia, resulting in root nodulation and nitrogen fixation (Oldroyd, 2013). In addition, rhizodeposition also functions as a chemical signal for the establishment of inter–root or root–microbe interactions (Jones et al., 2009). Therefore, different hosts, holding different gene sets, will trigger different responses to the same inoculant. Moreover, the same host will release different root exudates depending on the soil nutrient and microbial environment. All these examples represent the cyclic feedback between all the components of this ecosystem (plant–soil–microbes). This likely explains why field microbial inoculants fail to persist for long periods (Finkel et al., 2017). Moreover, the soil ecosystem plays a key role on the establishment of root microbiome (Edwards et al., 2015; Zarraonaindia et al., 2015), which means, that even if the inoculant survives within the soil community, it is not guaranteed that it would colonize the plant host.

**FIGURE 1:**
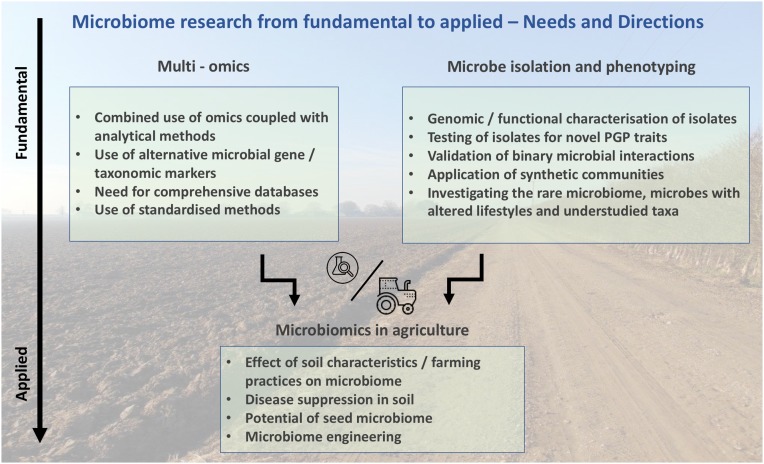
Potential of microbiome research; connecting fundamental microbiomics with applied microbiomics in agriculture.

Any benefits mediated by microbes observed under controlled conditions will ultimately need to be operative in the field. This implies their persistence in the field over time and successful plant colonization over a wide range of varying biotic, abiotic, and climate conditions. Therefore, finding single inoculants that can perform in such a variety of scenarios will be highly unlikely, which increases the need for the development of microbial precision agriculture mirroring the concept of human personalized medicine (Hamburg and Collins, 2010). In fact, the abundance of similarities between human gut and plant root microbiomes is striking and reveals the importance of the root microbiome in controlling plant fitness (Berendsen et al., 2012). Specifically, it has been shown that complex microbial inoculums can improve plant disease resistance and promote growth better than individual inoculums (reviewed in Finkel et al., 2017), highlighting the synergistic effects of a community. However, these findings still need to consider the soil context to address their potential use as soil amendments. In addition, a deeper understanding of a microbe’s function within a community and within a host would require functional studies where the ecosystem is challenged with different conditions (e.g., temperature, light, humidity). Those studies would validate their community interactions and their beneficial or detrimental outcome for plants as a prerequisite to justify for further field experiments (**Figure 1**).

### Addressing Field-Based Microbiome Studies

The immense microbial biodiversity in soil is regulated at very different scales, for example changes in soil texture, biotic interactions or plant root exudates have enormous effects down to the smallest (microbial) scales. Variations in the physical and chemical properties of soil, such as pH, nutrient distribution or water retention, have effects on soil biodiversity on the medium (field) scales with soil pH as a suggested major driver of microbial diversity (Fierer and Jackson, 2006) linking microbial community structure with soil nutrient availability and cycling (Li et al., 2017). Finally, at larger scales, geo-localization and climate might play more relevant roles on controlling soil biodiversity (Bardgett and van der Putten, 2014).

The existence of disease suppressive soils is living proof that microbial communities cannot just promote plant growth (Chaparro et al., 2012; Van Der Heijden et al., 2008) but also provide protection against plant pathogens (Alabouvette, 1986; Andrivon, 1994; Shiomi et al., 1999). Disease suppression can be due to competition with native soil fauna (general suppression) or to the presence of specific subsets of microbes (specific suppression). These soil protection strategies are comparable to immunity strategies of animals (Raaijmakers and Mazzola, 2016). As common to all complex ecosystems, general suppression is also common to all soils. Specific suppression, in turn, is removed by soil pasteurization and can be transferred to other soils via soil transplants. Soils can lose their suppressiveness if non-host plants are grown and can be recovered if the susceptible host and pathogen are grown back in them (Wiseman et al., 1996; Berendsen et al., 2012; Raaijmakers and Mazzola, 2016).

The ability of plants to attract beneficial root microbes might represent a crucial strategy to survive under unfavorable environmental conditions. Several studies point out to the possibility of engineering microbiomes to control plant traits that can be used to increase and sustain plant production (Mueller and Sachs, 2015; Panke-Buisse et al., 2015; Herrera Paredes et al., 2018; Orozco-Mosqueda et al., 2018). Plants challenged with pathogens can recruit protective microbes in the rhizosphere and endosphere that can modulate the host immune responses (Berendsen et al., 2018). This strategy has been exploited to formulate bioorganic fertilizers that manipulate banana rhizosphere microbial structure and subsequently decrease the incidence of Panama disease (Xue et al., 2015). All these studies have demonstrated the feasibility to engineer plant microbiomes as a sustainable solution to increase yields as well as biotic and abiotic stress resistance.

Another recent breakthrough discovery highly relevant for microbiome research is the analysis of seed microbiomes. It is supposed to have been co-selected and evolved with the plant providing valuable traits that have driven and still drive plant evolution (Puente et al., 2009; Johnston-Monje and Raizada, 2011; Turner et al., 2013a; Bouffaud et al., 2014; Delaux et al., 2014; Hardoim et al., 2015). Seed microbiomes seem to consist of a limited range of microbial species and this restricted number is probably due to the requirement of these species to survive all seed developmental processes, even the most extreme such as desiccation (Truyens et al., 2015). These studies are in accordance with recent experiments that have shown the possibility to transfer the plant microbiota to the next generation (Mitter et al., 2017). All these findings have put plant microbial engineering and breeding at the forefront of sustainable agriculture (Wei and Jousset, 2017).

To ultimately bridge the lab-field gap, we need to take into account that field experiments (in contrast to glasshouse experiments) are subject to agricultural practices, and these have a significant influence on microbiomes and microbial diversity. According to the UN, sustainable land management practices such as (i) crop rotation, intercropping and use of local plant varieties, (ii) tillage and organic farming should be re-introduced to minimize land degradation (Sanz, 2017). However, it is not clear how these practices can be re-introduced in agricultural systems of the developed world whilst still sustaining or increasing crop production. These practices also have a major contribution to microbiome community structure and function (Oberson et al., 1993; Mäder et al., 2002; Dossa et al., 2012; Debenport et al., 2015; Reganold and Wachter, 2016; Hartman et al., 2017, 2018; Wang Y. et al., 2017). Therefore, they might be important to consider when designing microbiome field experiments or testing commercial field applications of microbial inoculants.

#### Crop Rotation, Intercropping, and Use of Local Plant Varieties

Some ancient agricultural practices started to become less important around the 1940s, since monocropping and synthetic fertilizer applications significantly increased crop yields. These massive agricultural changes were part of the Green Revolution with the intention of feeding an increasingly growing human population. Together with the development of input-intensive agricultural systems for various single cash crops, other agricultural practices were no longer practical in developed countries. However, under the current global scenario of land degradation, fertilizer shortage or global warming, developing sustainable agriculture solutions face the challenge of feeding the still growing human population with minimal ecological and economic impact.

Land management has significant impacts on soil and root microbial community structure and stability and consequently on microbiome-associated functions (Hartman et al., 2017, 2018). Crops grown in monoculture or short rotations often suffer yield decline, due to an enrichment of pathogenic relative to beneficial microbes (Bennett et al., 2012; Hilton et al., 2013; Santhanam et al., 2015). In response, in a field setting, inoculation with native root-associated bacterial isolates can significantly decrease the emergence of diseases associated with continuous cropping (Santhanam et al., 2015) illustrating the potential for employing local microbial resources to increase plant yield and fitness in sustainable agriculture. As mentioned earlier, since soil disease suppression is lost when a different host plant is grown, this property also seems to be directly related to continuous cropping of the same species. These two opposite outcomes for agricultural production do not only reflect the enormous impact that plant hosts have on the soil microbiome, but also how the latter can impact on plant species that can successfully colonize an environment in natural conditions.

In addition to general edaphic factors, different plant species (Rovira, 1969), plant ecotypes (Micallef et al., 2009), or even different locations (microenvironment) of a root system (Pinton et al., 2007) result in the release of distinct root exudates. Therefore, soil microbial communities are shaped differently depending on the plant species grown. Intercropping was an ancient agricultural practice that was abandoned due to the development of modern intensive agricultural systems. However, intercropping is still a common practice in developing countries, where different plant species are grown in close proximity. Intercropping experiments performed in the Sahel region (Africa) have shown that this practice increases crop yield, soil organic carbon levels and community diversity of both bacteria and fungi (Debenport et al., 2015). Moreover, the co-cultivation with indigenous shrubs improves soil quality and N conservation (Dossa et al., 2012) highlighting the importance of using local species that have already adapted strategies to exploit the natural resources of an ecosystem. Intercropping has been suggested as an alternative for sustainable agriculture production. However, for it to become a common practice in developed countries, multiple challenges would need to be addressed, such as the development of cropping systems adapted to this agricultural practice.

In terms of microbiome research, more studies are required to comprehend how different cropping practices have such a relevant impact in the soil microbiota and whether both cropping practices and microbiome engineering could contribute to sustainable agriculture in the long term.

#### Tillage and Soil Farming

Land tilling is extended in modern agriculture since it minimizes weed growth and creates a seedbed that is adapted to the machinery commonly used in the field. Since the introduction of plant growth regulators in the 1940s (Bagavathiannan and Davis, 2018), no-tillage systems have been explored as a practice in conservation agriculture. However, no tilling systems require the use of cover crops and especially higher amounts of herbicides, which puts off many consumers and farmers. In turn, this practice minimizes soil particle disturbance, increases organic carbon soil content and enhances soil aggregation as well as water infiltration (Álvaro-Fuentes et al., 2008; Hobbs et al., 2008; Li et al., 2017; Wang Y. et al., 2017). Long-term no tillage and organic input management practices impact soil pH (being slightly higher in organic systems) (Mäder et al., 2002) and nutrient flux from the soil matrix to the soil solution. In terms of microbiome research, no-tilling and organic farming practices correlate with increases in soil microbial diversity, biomass and microbial community stability (Oberson et al., 1993; Mäder et al., 2002; Reganold and Wachter, 2016; Wang Y. et al., 2017). These positive effects on the soil microbiota are likely due to the increase of organic matter (acting as food resources for the microbial community), the decrease of physical perturbations (Wang Y. et al., 2017) and the increase in soil aggregate stability (Siegrist et al., 1998).

## Conclusion

The generation of microbial communities with customized (beneficial) activities has the potential to serve as a powerful approach to enhance sustainable agricultural production by increasing crop health, through combatting plant diseases and reducing the application of fertilizers. To reach this goal a fundamental understanding regarding the functioning of the plant microbiome through microbe–microbe and plant–microbe interaction is required, as well as a deeper understanding of the soil microbial community structure over time (long-term studies) and its plasticity and response to the environmental changes. Also, since individual microbes are key for the regulation of microbial community structure and stability, more comprehensive studies investigating community dynamics using these individual microbes and their soil microbial communities would assist in advancing the field. This knowledge could help to fully understand the impact that these keystone microbes have on crop yields, disease resistance and global nutrient cycles, but also to reveal strategies for microbiome engineering.

## Author Contributions

All authors listed have made a substantial, direct and intellectual contribution to the work, and approved it for publication.

## Conflict of Interest Statement

The authors declare that the research was conducted in the absence of any commercial or financial relationships that could be construed as a potential conflict of interest.
